# Synthesis of 2-(2-Hydroxyethoxy)-3-hydroxysqualene and Characterization of Its Anti-Inflammatory Effects

**DOI:** 10.1155/2020/9584567

**Published:** 2020-04-14

**Authors:** Kazunori Sasaki, Yuri Inami, Kenichi Tominaga, Hideo Kigoshi, Takashi Arimura, Hiroko Isoda

**Affiliations:** ^1^Alliance for Research on the Mediterranean and North Africa (ARENA), University of Tsukuba, 1-1-1 Tennodai, Tsukuba, Ibaraki 305-8572, Japan; ^2^Open Innovation Laboratory for Food and Medicinal Resource Engineering, National Institute of Advanced Industrial Science and Technology (AIST), University of Tsukuba, 1-1-1 Tennodai, Tsukuba, Ibaraki 305-8572, Japan; ^3^Interdisciplinary Research Center for Catalytic Chemistry, National Institute of Advanced Industrial Science and Technology (AIST), AIST Tsukuba Central 5-2, Tsukuba, Ibaraki 305-8565, Japan; ^4^Faculty of Pure and Applied Sciences, University of Tsukuba, 1-1-1 Tennodai, Tsukuba, Ibaraki 305-8571, Japan; ^5^Graduate School of Pure and Applied Sciences, University of Tsukuba, Tsukuba, Ibaraki 305-8571, Japan; ^6^Faculty of Life and Environmental Sciences, University of Tsukuba, 1-1-1 Tennodai, Tsukuba, Ibaraki 305-8572, Japan

## Abstract

Squalene (SQ), a natural precursor of many steroids, can inhibit tumor progression and decrease serum cholesterol levels. However, it is difficult to discern the effect of highly active molecules in the treatment of diseases because not enough active compounds reach the site of pathology in crowded biosystems. Therefore, it is necessary to design artificial probes that work effectively within crowded systems. In this study, to facilitate cell penetration, the ethylene glycol moiety (used as a probe) was chemically added to SQ to form 2-(2-hydroxyethoxy)-3-hydroxysqualene (HEHSQ). HEHSQ was prepared from 2,3-epoxysqualene and characterized by R_f_, FT-IR, ^1^H NMR, ^13^C NMR, and high-resolution mass spectrometry. We then evaluated the anti-inflammatory effects of SQ and HEHSQ on lipopolysaccharide- (LPS-) stimulated RAW264.7 murine macrophages. To determine the effect of SQ and HEHSQ on the viability of RAW264.7 cells, an MTT assay was performed. To quantify the anti-inflammatory effect of SQ and HEHSQ, we measured nitric oxide (NO) production, gene expression, and secretion of the proinflammatory cytokine tumor necrosis factor *α* (TNF-*α*) and chemokine C-C motif chemokine 2 (CCL2) in LPS-stimulated RAW264.7 cells using an *in vitro* inflammatory model. 2,3-Epoxysqualene was prepared according to a reported methodology. The reaction of 2,3-epoxysqualene and ethylene glycol in 2-propanol produced 49% HEHSQ. MTT results showed that 10 and 100 *µ*g/mL HEHSQ treatment decreased cell viability, whereas SQ treatment (1–100 *µ*g/mL) did not have any effect on viability. SQ (100 *µ*g/mL) and HEHSQ (1 *µ*g/mL) treatment significantly reduced the production of LPS-stimulated NO and decreased the expression and secretion of proinflammatory TNF-*α* and CCL2. Therefore, our results suggested that the anti-inflammatory effects of HEHSQ are 100 times higher than that of unmodified SQ. To the best of our knowledge, this study has demonstrated for the first time that HEHSQ can be potentially used as a safe alternative treatment to anti-inflammatory drugs.

## 1. Introduction

Inflammation is a protective response against trauma, infection, and tissue injury [1]. Acute inflammation usually subsides in a short while. However, if acute inflammation persists or the agent causing the inflammation is not eliminated, then acute inflammation can progress to a chronic stage. Chronic inflammation is a long-term phenomenon, causing tissue destruction and organ dysfunction. Furthermore, chronic inflammation is associated with various diseases including atherosclerosis, cardiovascular diseases, and arthritis [2]. Chronic inflammation is considered an underlying cause of several diseases, and thus, it is important to prevent such inflammation. Macrophages play a critical role during inflammation. Following tissue injury or infection first, the responder macrophages are activated and exhibit an inflammatory phenotype [2]. Activated macrophages produce numerous inflammatory mediators, such as cytokines (tumor necrosis factor-*α*: TNF-a), chemokines (CCL2), and nitric oxide (NO), which are highly toxic for microorganisms and can also be harmful to surrounding healthy tissues and lead to aberrant inflammation [3, 4]. For example, excessive or chronic NO production reacts with superoxide anions to become peroxynitrite, which is a very aggressive-free radical species, leading to the induction of mitochondrial dysfunction and cytotoxicity of the surrounding tissues [5–7]. And CCL2, which belongs to the C-C class of chemokines, is a critical modulator of inflammation, regulating macrophage recruitment during wound healing, infections, and autoimmune diseases [8]. Therefore, chemokines are involved in a variety of inflammatory conditions, both acute and chronic [9]. Thus, modulation of macrophage activation could be a good strategy to prevent chronic inflammation. Today, nonsteroidal anti-inflammatory drugs (NSAIDs) are the most commonly prescribed drugs worldwide. These display anti-inflammatory and analgesic effects; however, long-term use of NSAIDs causes undesirable side effects including gastrointestinal irritation, high blood pressure, and liver problems [10]. Therefore, complementary medicine that has anti-inflammatory properties and is derived from natural products has gained attention.

Squalene (SQ), a natural precursor of many steroids, is so named because of its presence in shark liver oil. It is also prepared from the microalgae *Aurantiochytrium* [11]. Because it is not toxic to humans, SQ has been extensively used as a dietary supplement [12, 13], an adjuvant in therapeutic applications [14], and for alleviating oxidative stress [15]. Moreover, our previous study revealed that the high SQ-producing microalgal strain (*Aurantiochytrium*) had an anti-inflammatory effect on lipopolysaccharide- (LPS-) stimulated mouse macrophage RAW264.7 cells in an *in vitro* inflammatory model [16]. Unfortunately, to date, attempts to improve the pharmacological effects of SQ have not met with much success. One of the factors limiting the pharmacological activity of SQ is its insolubility in polar solvents such as methanol, tetrahydrofuran, and dimethyl sulfoxide (DMSO); therefore, it cannot permeate cell membranes and reach tissues in a crowded environment.

In general, however, cells and tissues of living organisms make up a multimolecular system wherein diverse types of molecules get compartmentalized. Therefore, it is not often possible to use highly active molecules in the treatment of diseases, such as cancer, because enough active compounds cannot reach the site of pathology in crowded biosystems. Therefore, to improve pharmacological activity, artificial probes must be designed that effectively work within the crowded tissue architecture.

In the present study, we enhanced the pharmacological activity of SQ by increasing its bioavailability. The ethylene glycol moiety (used as a probe) was selectively introduced into SQ, creating HEHSQ, to facilitate tissue permeability. Also, we investigated the anti-inflammatory effect of SQ and HEHSQ on LPS-stimulated RAW264.7 murine macrophages.

## 2. Materials and Methods

### 2.1. Characterization of Squalene

All solvents used in the study were of reagent-grade quality and used without further purification unless otherwise noted. 2,3-Epoxysqualene was prepared according to a previously described methodology [17]. All column chromatography was undertaken using Merck silica gel 60 as the solid support.

HEHSQ was characterized by R_f_, FT-IR, ^1^H NMR, ^13^C NMR, and high-resolution mass spectrometry. The infrared (IR) spectrum was measured on a JASCO FT-IR-4100. NMR spectra were recorded on a Bruker Avance-400 spectrometer operated at 400 MHz and a JEOL RESONANCE ECX-100 spectrometer operated at 100 MHz at room temperature (20°C) in the Fourier transform mode. ^1^H NMR spectra were reported in *δ* units, parts per million (ppm), and calibrated relative to the signal for residual chloroform (7.26 ppm) in chloroform-d_1_ (CDCl_3_). ^13^C NMR data were reported in ppm relative to CDCl_3_ (77.16 ppm) and obtained with 1H decoupling. High-resolution mass spectrometric analyses were undertaken using an electrospray ionization time of flight based as a reserpine (*m/z* 609.2812) matrix on a JEOL JMS-100CS instrument.

### 2.2. Sample Preparation for *In Vitro* Study

SQ was purchased from Fujifilm Wako Co, Ltd. (Tokyo, Japan). SQ and HEHSQ were dissolved in DMSO to obtain the respective stock solutions, which were then diluted in cell culture medium for the *in vitro* experiment.

### 2.3. Preparation of LPS

LPS (*Escherichia coli* O111:B4) was purchased from EMD Millipore Co. (Billerica, MA, USA). A total of 5 mg of LPS was dissolved in 2 mL of phosphate-buffered saline without divalent cations (PBS (−)) and stored at −80°C in the dark until subsequent use.

### 2.4. Culture of RAW264.7 Cells

Murine macrophage-like RAW264.7 cells were purchased from RIKEN BioResource Center (RCB0535, RIKEN BRC, Tsukuba, Japan). RAW264.7 cells were cultured in Dulbecco's modified Eagle medium supplemented with 10% heat-inactivated fetal bovine serum and penicillin-streptomycin at 37°C in a humidified incubator containing 5% CO_2_. Cells were seeded onto 96-well plates at a density of 2.0 × 10^5^ cells per well and were incubated at 37°C for 24 h.

### 2.5. 3-(4,5-Dimethylthiazol-2-yl)-2,5-diphenyltetrazolium Bromide (MTT) Assay

Cell viability and mitochondrial activity were determined using the MTT assay to check the cytotoxicity of SQ (1, 10, and 100 *µ*g/mL) and HEHSQ (1, 10, and 100 *µ*g/mL). RAW264.7 cells were seeded at 2 × 10^5^ cells/mL in 96-well plates (BD BioCoat, USA) and incubated for 24 h. Then, the cells were treated with SQ or HEHSQ for 24 h. A solution of 5 mg/mL MTT dissolved in PBS was added (10 *µ*L/well) and incubated for another 24 h. The resulting MTT formazan was dissolved in 100 *µ*L of 10% sodium dodecyl sulfate (w/v), and the absorbance was measured using a microtiter plate reader (Dainippon Sumitomo Pharma Co., Ltd., Japan). The absorbance values were normalized to that of the culture medium, and viability was calculated as a percentage (%) of the untreated cells.

### 2.6. Measurement of NO Production

To evaluate the effect of SQ and HEHSQ on NO production in LPS-stimulated RAW264.7 cells, the Griess reaction was used following the methodology described in a previous study [16]. Briefly, RAW264.7 cells were seeded at 2 × 10^5^ cells/mL in 96-well plates (BD BioCoat) and incubated for 24 h. The cells were treated with SQ (1, 10, and 100 *µ*g/mL) or HEHSQ (1, 10, and 100 *µ*g/mL) at 37°C for 24 h. After the treatment, LPS solution (final concentration: 1 *µ*g/mL) was added to each well and incubated for 12 h at 37°C. Then, the cell supernatant was mixed at a 1 : 1 ratio with the Griess reagent (1% sulfanilic acid and 0.1% N-(1-naphthyl) ethylene diamine dihydrochloride in 2.5% phosphoric acid). After 10 min incubation in the dark, the absorbance was measured at 540 nm using a microtiter plate reader (Dainippon Sumitomo Pharma Co., Ltd., Japan), and nitrite concentration was determined by dilution of sodium nitrite (NaNO_2_, Fujifilm Wako Co, Ltd., Tokyo, Japan) as a standard.

### 2.7. RNA Isolation from RAW264.7 Cells

RAW264.7 cells were seeded at 3.7 × 10^5^ cells/mL in a 10 cm^2^ dish (BD BioCoat) and incubated at 37°C for 24 h. The RAW264.7 cells were then treated with SQ (100 *μ*g/mL) or HEHSQ (1 *μ*g/mL) at 37°C for 24 h. After the treatment, LPS solution (final concentration: 1 *μ*g/mL) was added to each well and incubated for a further 12 h at 37°C. Total RNA was isolated using ISOGEN (Nippon Gene, Tokyo, Japan) following the manufacturer's instructions as per a previous study [16].

### 2.8. Measurement of Proinflammatory Cytokine and Chemokine Using Real-Time Reverse Transcription Polymerase Chain Reaction (RT-PCR)

Real-time RT-PCR was performed to evaluate the effect of SQ or HEHSQ on proinflammatory cytokine and chemokine expression in RAW264.7 cells. The TaqMan probe (Thermo Fisher Scientific, USA) was used for the quantification of gene expression. Using a superscript III reverse transcriptase kit (Thermo Fisher Scientific), a complementary DNA (cDNA) solution was synthesized following the manufacturer's instructions. For quantification of transcript amounts, TaqMan real-time RT-PCR amplification reactions were performed using the Applied Biosystems 7500 Fast Real-Time System (Thermo Fisher Scientific). All primer sets and the TaqMan Universal PCR Master Mix were obtained from Thermo Fisher Scientific. Specific primers for actin beta (Mm02619580_g1), tumor necrosis factor *α* (TNF-*α*) (Mm00443258_m1), C-C motif chemokine 2 (CCL2) (Mm00441242_m1), and iNOS (Mm00440502_m1) were used.

### 2.9. Enzyme-Linked Immunosorbent Assay (ELISA) to Measure TNF-*α* and CCL2

The effects of SQ or HEHSQ on cytokine production in RAW 264.7 cells were determined by ELISA. In the assay, cells (3.7 × 10^5^ cells/mL) were seeded in a 10 cm^2^ dish (BD BioCoat) and treated with SQ (100 *μ*g/mL) or HEHSQ (1 *μ*g/mL) at 37°C for 24 h. After the treatment, LPS solution (final concentration: 1 *μ*g/mL) was added to each well and incubated for 12 h at 37°C. The cell supernatants were collected by centrifugation at 1000 ×*g* for 5 min, and the levels of TNF-*α* and CCL2 were determined using commercial ELISA kits (Proteintech Group, Japan) according to the manufacturer's instructions.

### 2.10. Statistical Analysis

All results are expressed as mean ± standard deviation (SD), and statistical evaluation was performed using the one-way ANOVA followed by *post hoc* Ryan-Einot-Gabriel-Welsch multiple range test. Differences were considered statistically significant when the *P* value was less than 0.05.

## 3. Results

### 3.1. Preparation and Identification of 2-(2-Hydroxyethoxy)-3-hydroxysqualene

**Figure 1:**
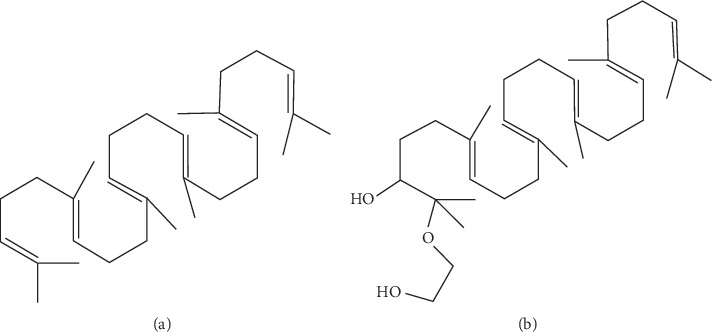
Structure of (a) 2,3-epoxysqualene and (b) 2-(2-hydroxyethoxy)-3-hydroxysqualene.

2,3-Epoxysqualene (Figure 1(a)) (31 mg, 71 mmol) and ethylene glycol (2.4 mL, 43 mmol) were dissolved in 2-propanol (9.6 mL). The mixture was heated to 80°C for 6 h while being stirred and then cooled to room temperature. H_2_O (15 mL) and ethyl acetate (30 mL) were added, and the organic layer washed with saturated brine and H_2_O and dried over Na_2_SO_4_. The solvent was evaporated following column chromatography on silica gel, eluting with hexane:ethyl acetate (5 : 1) produced HEHSQ (Figure 1(b)) (17 mg, 49%) as a colorless oil.

 
*R*_*f*_ = 0.08 (hexane:ethyl acetate = 4 : 1).
 
*IR* (CHCl_3_): 3451, 2977, 2932, 2877, 1452, 1381, 1219, 1083, 909, 670 cm^−1^.
 
^1^*H NMR* (400 MHz, CDCl_3_): *δ* 5.20–5.08 (m, 5H), 3.75–3.68 (m, 2H), 3.55–3.46 (m, 3H), 2.29 (m, 1H), 2.12–1.95 (m, 16H), 1.68 (s, 3H), 1.62 (s, 3H), 1.60 (br s, 12H), 1.52–1.37 (m, 5H), 1.15 (s, 3H), 1.14 (s, 3H) ppm.
 
^13^*C NMR* (100 MHz, CDCl_3_): *δ* 135.1, 135.1, 134.9, 134.8, 131.2, 124.7, 124.4, 124.3, 124.3 (2C), 77.8, 76.0, 62.4, 62.2, 39.7, 39.7 (2C), 36.8, 29.7, 28.6 (2C), 26.7, 26.7, 26.6, 25.7, 21.6, 19.8, 17.7, 16.0 (3C), 16.0 ppm.
 
*HRMS* (ESI): *m/z* 511.4131 (calculated for C_32_H_56_NaO_3_ [M + Na]^+^ 511.4127, Δ + 0.4 mmu).

### 3.2. Cytotoxic Effect of Squalene and 2-(2-Hydroxyethoxy)-3-hydroxysqualene on RAW264.7 Cells

**Figure 2:**
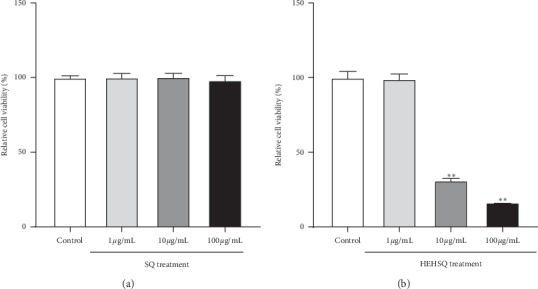
Effects of (a) squalene (SQ) and (b) 2-(2-hydroxyethoxy)-3-hydroxysqualene (HEHSQ) on the viability of RAW264.7 cells. RAW264.7 cells were treated with SQ or HEHSQ at 1, 10, and 100 *µ*g/mL for 24 h. Cell viability was measured by MTT assay. Values are expressed as the mean ± SD of triplicate experiments and are expressed relative to the percentage of control. The mean value that is significantly different from that of the control group is indicated as ^*∗∗*^*P* < 0.01.

To evaluate the cytotoxicity of SQ and HEHSQ, RAW264.7 cells were treated with SQ (1, 10, and 100 *µ*g/mL) or HEHSQ (1, 10, and 100 *µ*g/mL) for 24 h and cell viability was measured using the MTT assay. None of the SQ concentrations tested nor the 1 *µ*g/mL HEHSQ-treated cells showed any cytotoxic effects (Figures 2(a) and 2(b), respectively). Moreover, we also found that 1% DMSO- (100 *μ*g/mL) treated RAW264.7 cells showed no reduction of cell viability (Supplementary [Supplementary-material supplementary-material-1]). However, 10 and 100 *µ*g/mL of HEHSQ treatments showed a significant (*P* < 0.01) reduction in cell viability by 31.2 ± 1.3% and 16.5 ± 0.3%, respectively, compared with the untreated group (Figure 2(b)).

### 3.3. Effect of Squalene and 2-(2-Hydroxyethoxy)-3-hydroxysqualene on NO Production in LPS-Treated RAW264.7 Cells

**Figure 3:**
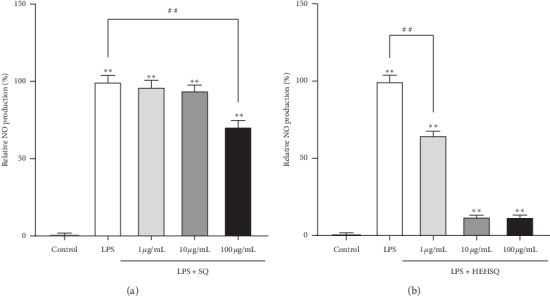
Effects of (a) squalene (SQ) and (b) 2-(2-hydroxyethoxy)-3-hydroxysqualene (HEHSQ) on LPS-stimulated NO production in RAW264.7 cells. Cells were treated with SQ or HEHSQ at 1, 10, and 100 *µ*g/mL for 24 h. Subsequently, cells were activated with LPS (1 *µ*g/mL) for 12 h. The amount of NO production was measured by the Griess reaction. Values are expressed as the mean ± SD of triplicate experiments and are expressed relative to the percentage of control LPS (−). The mean value that is significantly different from that of the control LPS (−) group is indicated as ^*∗∗*^*P* < 0.01. The mean value that is significantly different from that of the control LPS (+) group is indicated as ^##^*P* < 0.01.

NO, a chemical mediator produced by damaged tissues and inflammatory cells, is a part of a group of unstable radical moieties known as reactive oxygen species [18] and is one of the most important factors in the immune system [19]. In the present study, the effects of SQ and HEHSQ on NO production in LPS-stimulated RAW264.7 cells were evaluated. LPS- (1 *µ*g/mL) treated RAW264.7 cells showed the highest increase in NO production compared with the control cells. The treatment with SQ (100 *µ*g/mL) for 24 h significantly reduced LPS-stimulated NO production by approximately 30%, although 1 and 10 *µ*g/mL of SQ-treated cells did not show any significant change (Figure 3(a)). The 1 *µ*g/mL of HEHSQ-treated cells showed a significant reduction in NO production by approximately 35% compared with the LPS-stimulated cells (Figure 3(b)). Additionally, 10 and 100 *µ*g/mL HEHSQ-treated cells showed a significant decrease in NO production. However, this effect was most likely due to a decrease in cell viability. On the other hand, 1% DMSO- (100 *μ*g/mL) treated RAW264.7 cells did not show any reduction of NO production compared with the LPS-stimulated cells (Supplementary [Supplementary-material supplementary-material-1]).

### 3.4. Effect of Squalene and 2-(2-Hydroxyethoxy)-3-hydroxysqualene on Proinflammatory Cytokine and Chemokine Production in LPS-Treated RAW264.7 Cells

**Figure 4:**
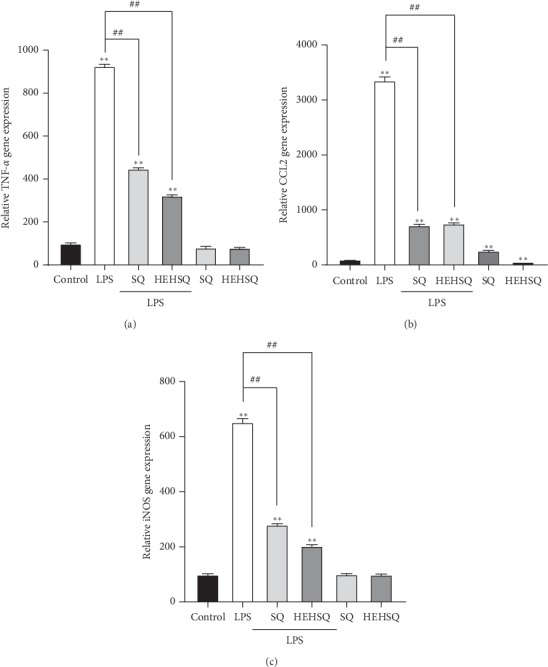
Effects of squalene (SQ) and 2-(2-hydroxyethoxy)-3-hydroxysqualene (HEHSQ) on gene expressions of (a) TNF-*α*, (b) CCL2, and (c) iNOS in RAW264.7 cells. RAW264.7 cells were treated with 100 *µ*g/mL SQ or 1 *µ*g/mL HEHSQ for 24 h. Following that, cells were activated with LPS (1 *µ*g/mL) for 12 h. After activation, the gene expressions of TNF-*α*, CCL2, and iNOS were evaluated using real-time RT-PCR. Values are expressed as the mean ± SD of triplicate experiments and are expressed relative to the percentage of control LPS (−). The mean value that is significantly different from that of the control LPS (−) group is indicated as ^*∗∗*^*P* < 0.01. The mean value that is significantly different from that of the control LPS (+) group is indicated as ^##^*P* < 0.01.

Proinflammatory cytokines and chemokines are mainly produced by activated macrophages and are involved in the modulation of the inflammatory reaction [20]. Although proinflammatory cytokines and chemokines are required as a defense mechanism for our body, the overproduction of proinflammatory cytokines can damage tissues and cause an abnormal immune response [4]. Thus, the regulation of proinflammatory cytokines, in turn, contributes to modulating excessive inflammatory responses. In the present study, to evaluate the effects of SQ and HEHSQ on LPS-stimulated inflammatory cytokine and chemokine production, TNF-*α*, CCL2, and iNOS gene expressions were measured by real-time RT-PCR. RAW264.7 cells were treated with 100 *µ*g/mL of SQ or 1 *µ*g/mL of HEHSQ for 24 h. After the treatment, RAW264.7 cells were stimulated with LPS for 12 h. The 100 *µ*g/mL of SQ-treated cells showed significantly lower (*P* < 0.01) TNF-*α* gene expression by approximately 50% (Figure 4(a)), CCL2 (Figure 4(b)) gene expression by approximately 80%, and iNOS (Figure 4(c)) gene expression by approximately 60% than that in LPS-stimulated RAW264.7 cells. The 1 *µ*g/mL of HEHSQ-treated cells also showed a significantly lower gene expressions of TNF-*α* by approximately 65% (Figure 4(a)), CCL2 (Figure 4(b)) by approximately 80%, and iNOS (Figure 4(c)) by approximately 70% than that in LPS-stimulated RAW264.7 cells. On the other hand, 1% DMSO- (100 *μ*g/mL) treated RAW264.7 cells did not show any reduction of gene expression of TNF-*α* (Supplementary [Supplementary-material supplementary-material-1]) and CCL2 (Supplementary [Supplementary-material supplementary-material-1]) compared with the LPS-stimulated cells.

### 3.5. Effect of Squalene and 2-(2-Hydroxyethoxy)-3-hydroxysqualene on Proinflammatory Cytokine and Chemokine Secretion in LPS-Treated RAW264.7 Cells

**Figure 5:**
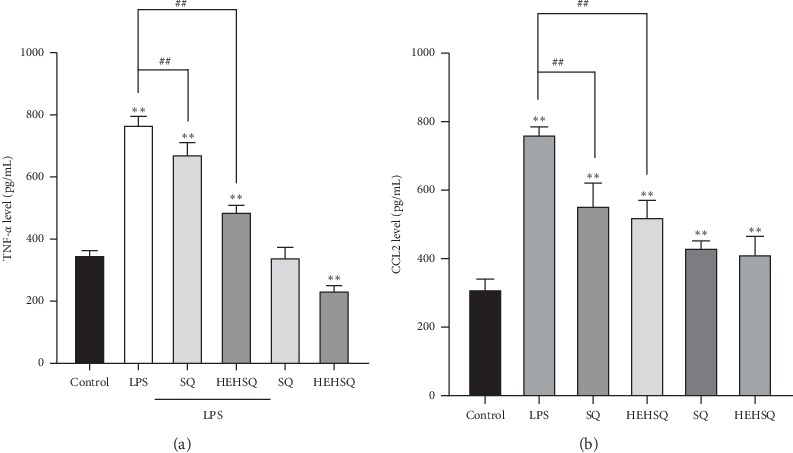
Effects of squalene (SQ) and 2-(2-hydroxyethoxy)-3-hydroxysqualene (HEHSQ) on the production TNF-*α* (a) and CCL2 (b) in RAW264.7 cells. RAW264.7 cells were treated with 100 *µ*g/mL SQ or 1 *µ*g/mL HEHSQ for 24 h. Following that, cells were activated with LPS (1 *µ*g/mL) for 12 h. After activation, the levels of TNF-*α* and CCL2 in the culture supernatant were evaluated using ELISA assays. Values are expressed as the mean ± SD of triplicate experiments and are expressed relative to the percentage of control LPS (−). The mean value that is significantly different from that of the control LPS (−) group is indicated as ^*∗∗*^*P* < 0.01. The mean value that is significantly different from that of the control LPS (+) group is indicated as ^##^*P* < 0.01.

ELISA assays were performed to quantify TNF-*α* and CCL2 secretion in the medium of RAW264.7 cells to confirm whether SQ or HEHSQ could regulate the inflammation in RAW264.7 cells. The MTT assay and NO assay showed that the optimal dose of SQ and HEHSQ were 100 and 1 *μ*g/mL, respectively. As shown in Figure 5, LPS-induced cells secreted significantly more TNF-*α* (770.7 ± 24.4 *μ*g/mL) and CCL2 (765.6 ± 19.8 *μ*g/mL) into the culture medium than untreated cells (351.4 ± 10.5 *μ*g/mL and 314.0 ± 26.1 *μ*g/mL, respectively). However, 100 *µ*g/mL SQ (675.2 ± 35.3 *μ*g/mL)- or 1 *µ*g/mL HEHSQ (490.4 ± 18.0 *μ*g/mL)-treated cells showed significantly lower (*P* < 0.01) TNF-*α* levels than that of the LPS-stimulated RAW264.7 cells (Figure 5(a)). Moreover, 100 *µ*g/mL SQ (558.0 ± 63.6 *μ*g/mL)- or 1 *µ*g/mL HEHSQ (524.7 ± 45.8 *μ*g/mL)-treated cells also inhibited CCL2 levels (Figure 5(b)).

## 4. Discussion

SQ, a biosynthesized triterpene hydrocarbon and a precursor for all steroids in animals and plants, is frequently used in the pharmaceutical and medical industries because it has several beneficial physiological effects such as the enhanced immune function, reduced serum cholesterol levels, protection from gamma rays, and suppression of tumor growth [12, 21–23]. In the present study, a molecular design was created to enhance the pharmacological activity of SQ in a crowded cellular environment. The introduction of the ethylene glycol moiety as a probe at the 2-position of SQ allows it to penetrate biological tissues because HEHSQ is highly soluble in methanol, tetrahydrofuran, and dimethyl sulfoxide, which are miscible with water. As to the preparative quantity of HEHSQ in the synthetic procedure from 2,3-epoxysqualene, it gives a small amount of HEHSQ, 17 mg (49% yield). However, in the future study, we plan to improve the synthetic method that can be used to prepare quantities up to around 2000 mg at a time in a 1-liter flask.

First, in the *in vitro* study, an MTT assay was performed to evaluate the cytotoxicity of SQ and HEHSQ. The MTT assay results showed that the 1 to 100 *µ*g/mL SQ treatment did not change the cell viability, thus suggesting that it may be difficult for SQ to enter cells. However, the HEHSQ treatment showed a significant decrease in cell viability at 10 and 100 *µ*g/mL concentrations. These results suggest that the addition of ethylene glycol to SQ, creating HEHSQ, increased the bioavailability of SQ.

In the present study, we evaluated the anti-inflammatory effects of SQ and HEHSQ on LPS-stimulated RAW cells in an *in vitro* inflammatory model. LPS triggers several inflammatory reactions by binding to its specific receptor, toll-like receptor 4 (TLR4) in macrophages [24], and hence, it is widely used as an inflammatory stimulator. Once LPS binds to TLR4, several downstream signaling pathways are activated, resulting in the production of inflammatory substances including proinflammatory cytokines and NO. Normally, NO is known to exert different actions in various biological processes, including immune defense response and inflammation [5]. In the present study, we found that LPS-treated RAW264.7 cells increased NO production. Although low concentrations of SQ (1 and 10 *µ*g/mL) did not affect NO production, high SQ concentration (100 *µ*g/mL) showed lower NO production than that of the LPS-stimulated RAW cells. Additionally, NO production in RAW cells treated with low concentration HEHSQ (1 *µ*g/mL) was significantly lower than that in the LPS-stimulated RAW cells.

TNF-*α*, one of the major proinflammatory cytokines, is released from macrophages in response to inflammation and induces vasodilation and loss of vascular permeability, which facilitates immune cell infiltration including lymphocytes, neutrophils, and monocytes [25]. TNF-*α* helps recruit immune cells into sites of inflammation and hence promotes inflammatory responses. It has been reported previously that excess amounts of TNF-*α* play a pathological role in several diseases including inflammatory bowel diseases, rheumatoid arthritis, and asthma [25]. Similar to cytokines, the chemokines (e.g., CCL2) play key roles in the inflammation process [26]. Chemokines, a family of small soluble proteins that regulate cell migration via the formation of concentration gradients, exhibit a high degree of conservation between mice and humans and have long been recognized as critical mediators of immune cell trafficking during embryonic development, wound healing, and infection [27–29]. Thus, regulating the production of inflammatory mediators, such as TNF-*α* and CCL2, contributes to an anti-inflammatory effect. Moreover, preventing overproduction of inflammatory mediators shifts the balance towards the beneficial effects of chronic inflammation, and it may prevent several diseases. In the present study, the effect of SQ and HEHSQ on TNF-*α*, CCL2, and iNOS gene expressions (production) and TNF-*α* and CCL2 levels in the culture supernatant (secretion) were evaluated using LPS-stimulated RAW264.7 cells. Our results showed that LPS treatment significantly increased the expressions of TNF-*α*, CCL2, and iNOS compared with untreated RAW264.7 cells. Moreover, TNF-*α* and CCL2 levels in the culture supernatant of LPS-treated cells were also higher than that of untreated cells. However, SQ (100 *µ*g/mL) and HEHSQ (1 *µ*g/mL) treatments significantly lowered the expression and secretion of TNF-*α* and CCL2 upon LPS stimulation. Therefore, our results indicated that the ethylene glycol modification (HEHSQ) enhanced the anti-inflammatory effect of SQ by 100 fold compared with unmodified SQ. Our results suggest that HEHSQ, which shows anti-inflammatory properties at low concentrations (1/100 concentration of SQ), could be used in dietary supplements, cosmetics, and functional foods for the prevention and treatment of chronic inflammatory diseases.

In this study, we have uncovered the anti-inflammatory effects of HEHSQ; however, the underlying molecular mechanism effect remains unclear. We used LPS-stimulated RAW264.7 cells to evaluate the anti-inflammatory effect. LPS is a well-known endotoxin that initiates inflammatory responses. LPS binds to TLR4 and effects several adaptor proteins such as toll-IL-1 receptor domain-containing adaptor protein, TNF-receptor-activated factor 6, and transforming growth factor *β*-activated kinase 1. These receptor and adaptor proteins transmit signals to several pathways, such as nuclear factor-kappa B (NF-*κ*B), one of the key regulators of inflammation and mitogen-activated protein kinase (MAPK) signaling [30]. NF-*κ*B signaling can be activated by various stimulators such as inflammatory cytokines, LPS, microorganisms, and ultraviolet light. Activated NF-*κ*B signaling induces an increase in the expression of proinflammatory cytokines and NO synthase [31]. MAPK signaling is also another major pathway involved in the inflammatory response. Transforming growth factor *β*-activated kinase 1 activates MAPK kinases, and they subsequently phosphorylate extracellular signal-regulated kinase, p38, and c-Jun N-terminal kinase. Moreover, phosphorylation of activator protein-1, ETS Like-1 protein Elk-1, and transcription factors initiate the transcription of inflammatory genes [32]. In the future, the effect of HEHSQ on NF-*κ*B and MAPK signaling, which are the key mediators of inflammatory signaling, should be evaluated.

## 5. Conclusions

In conclusion, to the best of our knowledge, our study provides the first evidence that SQ decreases the inflammatory mediators NO, TNF-*α*, and CCL2. Moreover, we also showed that the anti-inflammatory effects of HEHSQ are 100 times more potent than those of natural SQ. Our results in this study could be a great contribution to local pharmacological applications, mainly for the handicap of higher cell toxicity. The results presented here suggest that HEHSQ may be potentially used as a safe alternative treatment to anti-inflammatory drugs. Therefore, we recommend the consumption of foods containing SQ, such as shark liver oil and olive oil, because oral administration of SQ is well absorbed (60–85%) in humans [32] and minimize the SQ solubility and bioavailability problems of several pharmacological activities.

## Data Availability

The data used to support the findings of this study are included within the article.

## Abbreviations

CCL2: C-C motif chemokine 2
cDNA: Complementary DNA
LPS: Lipopolysaccharide
MAPK: Mitogen-activated protein kinase
MTT: 3-(4,5-Dimethylthiazol-2-yl)-2,5-diphenyltetrazolium bromide
NF-*κ*B: Nuclear factor-kappa B
NO: Nitric oxide
NSAIDs: Nonsteroidal anti-inflammatory drugs
TNF-*α*: Tumor necrosis factor-*α*
TLR4: Toll-like receptor 4.

## Supplementary Materials

Supplementary Materials Supplementary figures provide data on the cytotoxic effects (Figure 1) and anti-inflammatory effects (Figure 2 and Figure 3) of DMSO as a vehicle.
