# Supplementary material for: Synthesis of 2-(2-Hydroxyethoxy)-3-hydroxysqualene and Characterization of Its Anti-Inflammatory Effects
Source: Biomed Res Int. 2020 Apr 14;2020:9584567. doi: 10.1155/2020/9584567 (PMC7180416; doi:10.1155/2020/9584567)
Supplement: Supplementary Materials — Supplementary figures provide data on the cytotoxic effects (Figure 1) and anti-inflammatory effects (Figure 2 and Figure 3) of DMSO as a vehicle. [file 9584567.f1.docx]

**Supplementary figures**

Sasaki et al.,

Figure S1

**Figure S1.** Effect of DMSO on the viability of RAW264.7 cells. RAW264.7 cells were treated with 1% DMSO for 24 h. Cell viability was measured by MTT assay. Values are expressed as the mean ± SD of triplicate experiments and are expressed relative to the percentage of control.

Figure S2

**Figure S2.** Effect of DMSO on LPS-stimulated NO production in RAW264.7 cells. Cells were treated with 1% DMSO for 24 h. Subsequently, cells were activated with LPS (1 ng/mL) for 12 h. The amount of NO production was measured by the Griess reaction. Values are expressed as the mean ± SD of triplicate experiments and are expressed relative to the percentage of control LPS (-). The mean value that is significantly different from that of control LPS (-) group is indicated as ** P < 0.01.

Figure S3

**Figure S3.** Effect of DMSO on gene expression of (A) TNF-α and (B) CCL2 in RAW264.7 cells. RAW264.7 cells were treated with 1% DMSO for 24 h. Following that, cells were activated with LPS (1 ng/mL) for 12 h. After activation, the gene expression of TNF-α and CCL2 were evaluated using real-time RT-PCR. Values are expressed as the mean ± SD of triplicate experiments and are expressed relative to the percentage of control LPS (-). The mean value that is significantly different from that of the control LPS (-) group is indicated as ** P < 0.01.
